# Dynamic Removal of Nickel (II) on *Elaeis guineensis* Waste Bed: Study of the Breakage Curve and Simulation

**DOI:** 10.3390/ijerph192416668

**Published:** 2022-12-12

**Authors:** Candelaria Tejada-Tovar, Angel Villabona-Ortíz, Ángel Darío González-Delgado

**Affiliations:** 1 Process Design, and Biomass Utilization Research Group (IDAB), Chemical Engineering Department, Universidad de Cartagena, Avenida del Consulado St. 30, Cartagena de Indias 130015, Colombia; 2 Nanomaterials and Computer-Aided Process Engineering Research Group (NIPAC), Chemical Engineering Department, Universidad de Cartagena, Avenida del Consulado St. 30, Cartagena de Indias 130015, Colombia

**Keywords:** adsorption, heavy metals, dose–response model, oil palm, agro-industrial waste, Aspen Adsorption

## Abstract

This research focused on the use of residual fiber from oil palm (*Elaeis guineensis*) for Ni (II) adsorption in a packed bed column. An analysis was conducted on the effect and statistical incidence of changes in temperature, adsorbent particle size, and bed height on the adsorption process. The results showed that particle size and bed height significantly affect the adsorption of Ni (II) ions, reaching adsorption efficiencies between 87.24 and 99.86%. A maximum adsorption capacity of 13.48 mg/g was obtained in the bed with a break time of 180 min. The Ni (II) adsorption in the dynamic system was evaluated by the analysis of the breakage curve with different theoretical models: Yoon–Nelson, dose–response, and Adams–Bohart; the dose–response model was the most appropriate to describe the behavior of the packed bed with an R^2^ of 84.56%. The breakthrough curve obtained from Aspen Adsorption^®^ appropriately describes the experimental data with an R^2^ of 0.999. These results indicate that the evaluated bioadsorbent can be recommended for the elimination of Ni (II) in aqueous solutions in a dynamic system, and the simulation of the process can be a tool for the scalability of the process.

## 1. Introduction

Currently, many types of research have detected the contamination of groundwater sources with various heavy metals as a result of anthropogenic activities or natural factors [1]. Nickel is a heavy metal naturally found in the environment [2]. Nevertheless, its presence has increased due to effluents from industrial sectors, such as pharmaceutical, paint, batteries, electroplating, paper, and oil refining [3]. Exposure to Ni (II) can cause problems to humans and the environment, as it is highly persistent, resistant to biodegradation, and can cause multiple diseases, such as skin or dermatitis problems; lung, bone, and nose cancer; and encephalopathy [4].

In this sense, studies have been developed to reduce the presence of heavy metals dissolved in water, applying processes such as chemical precipitation, ion exchange, membrane processes, electrodialysis, phytoextraction, ultrafiltration, and reverse osmosis [5]. These methods are not economical and have shortcomings such as the incomplete removal of metals, the high cost of reagents, energetic necessities, and the generation of toxic sludge [6]. Therefore, adsorption is currently recognized as an effective and economical method for the treatment of wastewater, highlighting the advantage that allows the use of materials that, in many cases, are considered agricultural or agro-industrial waste [7].

Biomaterials of vegetable origin are an attractive source for the preparation of bioadsorbents for the existence of functional groups such as OH, COO^−^, carbonyl, and CH_x_, since they comprise lignin, cellulose, hemicellulose, and pectin [8]. In particular, the African palm oil *(Elaeis guineensis*) extraction industry produces huge quantities of residues during its process. This includes palm bagasse and pit. The increase in oil production from oil palms generates about 500,000 tons of waste per year [9]. Even though the pit produced is used as fuel and fertilizer, the residual fiber is a waste product that has no use. Therefore, the utilization of this waste could mitigate its effects over the environment. Amid the several uses that could be given to the residual fiber of palm is its use as an adsorbent for wastewater treatment polluted with heavy metals; the treatment of wastewater seems attractive for the removal performance reported for similar waste [10]. The advantage of adsorbents prepared from waste is that they are low cost, in addition to being ideal as adsorbents because of their properties of large surface, high adsorption capacity, mechanical stability, compatibility, easy access, easy regeneration, cost-effectiveness, and simple and highly selective procedures [11].

An extensive type of biomaterials has been efficiently used to synthetize adsorbents with appropriate properties to adsorb Ni (II), such as biochar [12,13], Macauba waste [14], and zeolites [15]. When using coconut shells, it was found that increased flow causes a decrease in adsorption capacity, while increased bed height positively affects bed performance; a maximum bed adsorption capacity of 5.45 mg/g for a flow of 200 mL/min was achieved [16]. One study used bagasse of palm modified with citric acid, finding that the model of Thomas described the breakage curve with a removal efficiency of 92.58% m using a constant bed height of 5.5 cm, room temperature, and the experiments developed in a maximum time of 2.5 h in columns of a 6.6 cm diameter with a maximum capacity of 140 mL [17]. Thus, in this study, adsorption experiments were carried out in a continuous packed bed system using oil palm bagasse to determine the effect of biomass particle size, solution temperature, and bed height on the adsorption of Ni (II) ions present in the aqueous solution.

## 2. Materials and Methods

### 2.1. Materials

For the synthetic solution of Ni (II), nickel sulfate (NiSO_4_) of analytical grade was used as a reagent. Adsorption experiments were carried out on an acrylic column containing the flowing metallic solutions with a flow rate of 0.75 mL/, 100 mg/L, and pH 6 [18].

### 2.2. Methods

#### 2.2.1. Preparation of the Bioadsorbent

The residual palm fiber was collected as a reject product from a palm oil extraction plant located in the department of Bolívar, Colombia. The biomass was washed with deionized water, dried in the sun to a constant mass, and crushed in a blade mill. The size classification was carried out in an Edibon Orto Alresa shaker-type sieve machine, using meshes No 16, 18, 35, 45, and 70 [19]. The bioadsorbent was characterized by Scanning Electron Microscopy (SEM) and Energy Dispersive Spectrometry (EDS) analysis in order to study the morphology of the adsorbent and its elemental composition before and after adsorption, in a JEOL scanning electron microscope, model JSM 6490-LV [20].

#### 2.2.2. Continuous System Adsorption Experiments

Ni (II) solutions were used at 1000 mg/L. For the development of the adsorption experiments, a central experimental design composed of 2^3^ + rotatable star was followed, carried out in Statgraphics Centurion XVII, where the height of the bed (6.13, 30, 65, 100, 7, 124 mm), the particle size (0.13, 0.355, 0.5, 1, 1.2 mm) and the temperature (30, 40, 55, 70 and 80 °C) were varied to evaluate their effect on NI (II) removal efficiency [21].

Concentration measurements were determined after the adsorption tests with atomic absorption spectrophotometry at 232 nm [2]. The removal efficiency (RE) was determined using Equation (1) [22].

(1) $$RE\left(\%\right)= [\frac{C_{0}-C_{t}}{C_{0}}\times 100]$$

where $C_{t}$ is the final concentration of the Ni (II) ion at the end of the column in mg/L and $C_{0}$ is the initial concentration of the metal in mg/L, with 100 mg/L being the concentration used in all cases.

The results were analyzed in Statgraphics Centurion XVI software, determining the significant variables of the process through an analysis of variance ANOVA and the optimal conditions of the adsorption process for each metal/adsorbent system, with which the breakage curve and the model adjustments were made through nonlinear regression. The models that were used are summarized in Table 1. The data adjustment was defined based on the determination coefficient R^2^, which is the proportion of total variance explained by the regression.

The total adsorption capacity of the packed bed was determined using Equation (2).

(2) $$Q_{total}=\frac{Q}{1000m}\;\int_{0}^{t_{s}}C_{t}-C_{0}dt$$

where $Q_{total}$ is the adsorption capacity of the oil palm bagasse (mg/g); *C*_0_ and *C_t_* are the initial and final concentration of Ni (II) in fluid in mg/L; *t_s_* (min) is the time taken for the cake to make *C*_0_ = *C_t_*; *Q* is the flow rate in mL/min; and *m* is the mass of the adsorbent in the column (g) [24].

## 3. Results and Discussion

### 3.1. Characterization of the Bioadsorbent

**Figure 1:**
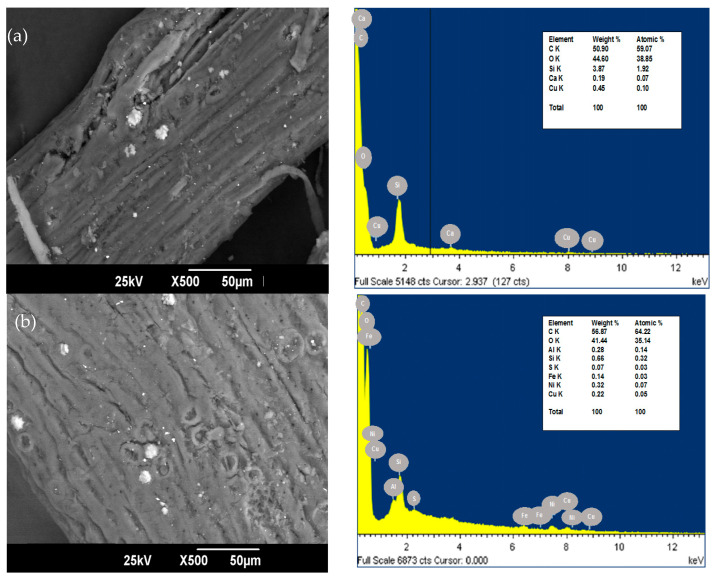
SEM of palm bagasse (**a**) before and (**b**) after Ni (II) adsorption with magnification ×500.

The SEM and EDS of oil palm bagasse before and after Ni (II) removal are presented in Figure 1. The bioadsorbent exhibits a rough and fibrous surface with the presence of mesopores, which coincides with what is reported by [25]. It was previously found that oil palm fiber has a homogeneous surface with a very porous texture [26]. After the adsorption process, softening of the grooves is present in the bioadsorbent, which can be credited to the retention of the metallic ion in the available sorption centers [27]. In addition, after the NI (II) removal process, the bioadsorbent was observed to have a higher zone of nodular particles with respect to the raw material. This could be on the grounds that the cellular structure may have enlarged after adsorption by the diffusion of the solution through the cellular structure of the material.

The EDS spectrum of carbon and oxygen, at 0.270 keV and 0.530 keV, respectively, contains the elements more abundant in the structure of the oil palm bagasse. This is because cellulose is the principal constituent of the bioadsorbent, and these elements mainly compound it [28]. After the adsorption, Ni (II) was found in the surface structure of the palm fiber in the characteristic peaks of high intensity at 1.69 keV. This suggests that bonds are formed between the ion under study and the active adsorption centers present in the bioadsorbent [29].

The difference observed in the palm bagasse composition before and after the adsorption of Ni (II) ions is because ions could interact with the bioadsorbent’s surface and react with the active centers, leading to the disappearance of elements such as Ca, the decrease in Cu and Si composition, and the appearance of minerals that structurally constitute plants such as S and Fe.

On the other hand, it has been suggested that Ni (II) adsorption mechanisms involve cation exchange, surface complexation, precipitation, co-precipitation, and diffusion. In the present study, it can be said that nickel adsorption could be controlled by ion exchange, complexation of the inner sphere, and coprecipitation on the surface of sugarcane bagasse, taking into account the possible formation of bidentate complexes of internal sphere with groups N-C=O/NH_3_, OH and C=O/C-O, which coincides with that reported by Leng et al. [30] for the alga *Ulva lactuca*.

### 3.2. Effect of Particle Size and Bed Height

From Table 2, it can be seen that the bed height, the particle size, and the interaction between the two have a noteworthy incidence on the process of Ni (II) adsorption on oil palm bagasse, as indicated by the *p*-value < 0.05. In the same sense, the F-ratio is a reliable criterion for distinguishing important factors from less important ones. Higher values for the calculated F-ratio indicate a greater influence of the factor on the outcome of the experiment. Taking the above into account, it can be said that the variables with the most significant effects are particle size and bed height, which is consistent with what is shown by the *p*-value. The above shows that these variables affect the interaction of ion binding with the active centers of the adsorbent or the formation of new active sites for adsorption.

**Figure 2:**
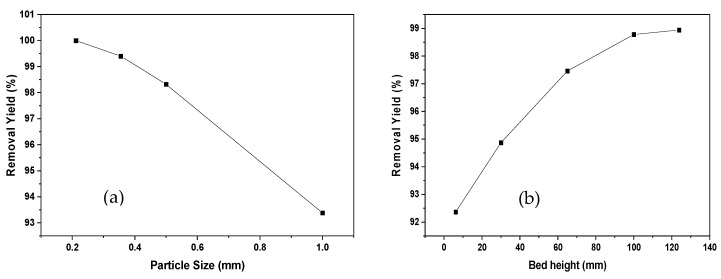
Effect of particle size (**a**) and bed height (**b**) of Ni (II) adsorption onto oil palm bagasse residue in a continuous system.

Figure 2 represents the effect of particle diameter and bed height on Ni (II) adsorption. An increase in the effectiveness of removal is observed due to the augmentation in bed height. This may be due to the increase in the residence time of the solution with the biomaterial under study, which represents a longer contact time; therefore, the metal has more time to emigrate into the solid phase [31].

Likewise, it can be said that a greater amount of adsorbent in the column offers additional junction sites, obtaining an extended mass transfer area [19]. For reduced bed depths, axial scattering phenomena prevail in the transport of the solute from the bulk, reducing the diffusivity; therefore, a decrease occurs in the removal efficiency [32]. As a result, the Ni (II) ions do not have sufficient time to be transferred into the adsorbent, abandoning the column before the process is complete. Regarding the particle size, it is observed that an increase from 0.212 to 1 mm causes a decrease of 7% in the removal efficiency, resulting in a slower pore diffusion rate due to a higher resistance to diffusion [33]. It has been documented that the smaller the particle size, the greater the surface area of contact, causing increased removal efficiency [34]. Similar observations were also determined by [33] in the study of the removal of cobalt (II) using sunflower biomass considering different experimental conditions.

### 3.3. Modeling the Breakage Curve

The Ni (II) adsorption breakage curve experiment at the optimal conditions obtained a temperature of 80 °C for the fluid, a particle size of 0.212 mm, and a 50.3 mm bed height. According to Equation (2), a *q_max_* of 13.48 mg/g was obtained. Table 3 shows different studies that report the evaluation of materials present in the adsorption of Ni (II) using a packed bed system. It is observed that the palm bagasse evaluated in this study is a good alternative for the removal of Ni (II) in a packed bed column, due to the bed’s adsorption capacity.

**Figure 3:**
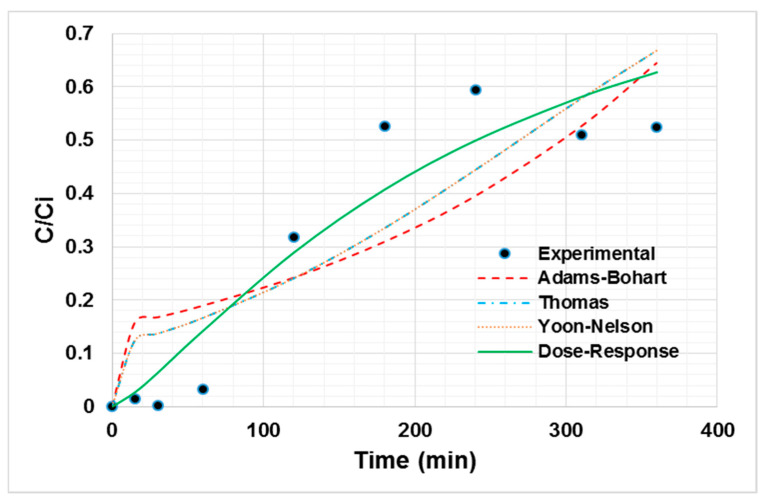
Non-linear adjustment of Ni (II) adsorption breakage curve models at 80 °C, a particle size of 0.212 mm, 100 mg/L of initial concentration, 0.75 mL/s of flow, and a bed height of 50.3 mm.

From the breakage curve of Ni (II) adsorption onto oil palm bagasse, it can be said that the greatest ability to retain the ion on the adsorbent materials lies in the first instances of contact with the bioadsorbent. In addition, it is observed in Figure 3 that the breakthrough point is near 3 h. The decreasing trend in the removal efficiency with the time is due to the occupation of active sites [41]. The experimental data were fitted to the Thomas, dose–response, Yoon–Nelson, and Adams–Bohart models, as shown in Figure 3, to determine the process characteristics. The fitting parameters are shown in Table 4.

From the non-linear regression, the best fitting dose–response model is established with an R^2^ of 84.56% [42]. The value of *q*_0_ calculated by the model is close to the experimental one, corroborating the good fit of the model [43]. According to the literature, for the bed height evaluated between 50 and 100 mm, the axial dispersion phenomena increase. This increases the diffusivity of the ions in the bed. Thus, the ions have enough time to diffuse in the adsorbent due to the high availability of adsorption sites, influencing the adsorptive capacity of the bioadsorbent [44]. Nevertheless, the empirical parameter “a” is not easy to link with a physical meaning or with the experimental conditions evaluated during our study, so it is difficult to scale the system using the dose–response model [45]. However, from the experimental data it can be said that the dispersion does not play a significant role in the current column system, considering the low inlet flow used, as well as the bed height.

In order to apply the adsorption process in a continuous system at industrial scale, the breakage time must be estimated, considering that it establishes the bed lifetime. In this study, it was demarcated as the time interval in which C_f_ > 50% C_i_, and the experimental breakage time was close to 180 min. Although in the present study such saturation was not reached after the operational time, it is established that in the study time the biomass can be implemented in the removal of Ni (II) [46].

**Figure 4:**
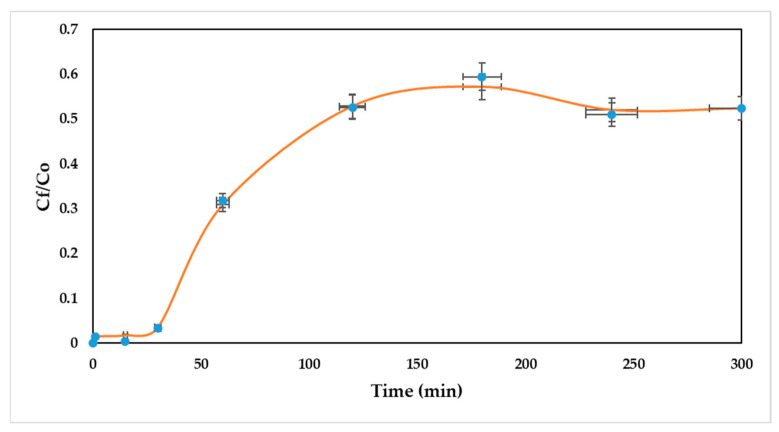
Comparison of simulated and experimental breakthrough curves. The error bars indicate the standard deviation between experimental and simulated data.

In this study, the adsorption of Ni (II) onto a packed bed of oil palm bagasse was simulated using the Linear Driving Force (LDF) as a kinetic model, and the Langmuir model for equilibrium description. The validation of the experimental data by simulation was carried out to select and assess the performance of the padding of the column. For this, errors were obtained with respect to the experimental data. The contrast of the experimental and simulated breakthrough curves in Aspen Adsorption^®^ is shown in Figure 4.

As can be seen, the simulated breakthrough curve accurately represents the experimental breakthrough curve for nickel (II) removal using a packed bed of oil palm bagasse. The above can be corroborated by the high value of R^2^ (0.999). Nevertheless, for the scaling to industrial processes, determining the effect of the dispersion coefficient of the column might be significant, as well as mass transfer coefficients, in order to explain the possible nonidealities at later times. Thus, the breakthrough obtained from Aspen Adsorption^®^ is considered as characteristic for the removal of Ni (II) ions onto a column packed with oil palm residues, showing good validation of the experimental results.

## 4. Conclusions

In the present study, the valorization of agro-industrial wastes from oil palm as an adsorbent of Ni (II) was studied in a packed bed continuous system, evaluating the temperature, bed height, and particle size effect on the removal process. From the superficial characterization of SEM-EDX, natural oil palm bagasse was found to have a rough and fibrous surface with mesopores. After the adsorption process, nodular particles were detected, and this was attributed to the enlargement of the cell structure after adsorption. This study concluded that the efficiency of the removal of Ni (II) ion was obtained between 87.24 and 99.86%, showing the affinity of the residual oil palm fiber biomaterial for the ion. The increase in bed height and the decrease in particle size had a positive effect on the process. The theoretical maximum removal for Ni (II) was 102.745% with the highest temperature (80 °C), smallest particle size (0.212 mm), and medium bed height (50.3 mm), and a breakage time of 180 min was obtained. The model that better adjusted the experimental data of the breakage curve was the dose–response model with an R^2^ of 84.56%. The breakthrough curve obtained from Aspen Adsorption^®^ appropriately describes the experimental data and is a useful tool to scale the adsorption process in a continuous system. These results indicate that the evaluated bioadsorbent can be recommended for the elimination of Ni (II) in aqueous solutions in a dynamic system, and the simulation of the process can be a tool for the scalability of the process and a facilitator for technology transfer.

## Figures and Tables

**Table 1:** Ni (II) adsorption breakage curve adjustment models [23].

Model	Equation	Parameters
Thomas	$\frac{C_{0}}{C}=\frac{1}{1+EXP [\frac{K_{th}}{Q}\;(q_{0}X-C_{0}V]}$	*K_th_* (mL/min^−1^ mg^−1^): constant of Thomas *q*_0_ (mg/g): maximum solute concentration in the solid phase *X* (g): the amount of adsorbent in the column *Q* (mL/min): flow rate *V* (L): effluent volume at operation time
Yoon–Nelson	$\frac{C}{C_{0}}=\frac{Exp\;\left(K_{YN}t-\tau K_{YN}\right)}{1+Exp\;\left(K_{YN}t-\tau K_{YN}\right)}$	*K_YN_* (min^−1^): proportionality constant *τ* (min): time to retain 50% of the initial adsorbate
Dose–response	$\frac{C}{C_{0}}=1-\frac{1}{1+{ [\frac{C_{0}*Qt}{q_{0}X}]}^{a}}$	*a*: model constant *q*_0_ (mg/g): maximum solute concentration in the solid phase *X* (g): the amount of adsorbent in the column *Q* (mL/min): flow rate
Adams–Bohart	$\frac{C}{C_{0}}=\frac{exp\left(K_{AB}C_{0}t\right)}{exp\left(\frac{K_{AB}\;N_{0}L}{v}\right)-1+exp\left(K_{AB}C_{0}t\right)}$	*K_AB_* (L/mg.min): model kinetic constant *N*_0_ (mg/L): maximum volumetric adsorption capacity *V* (cm/min): linear flow velocity *L* (cm): depth of the column bed *C*_0_ (mg/g): initial Ni (II) concentration *C_t_* (mg/L): final Ni (II) concentration *t* (min): residence time of the solution in the column

**Table 2:** ANOVA of nickel removal experimental data.

Source	Sum of Squares	F-Ratio	*p*-Value
A: Temperature	1.74	1.83	0.2247
B: Particle size	123.78	130.38	0.0000
C: Bed height	80.31	84.60	0.0001
AA	1.11	1.16	0.32
AB	2.51	2.65	0.15
AC	0.43	0.45	0.53
BB	1.39	1.47	0.27
BC	32.09	33.80	0.0011
CC	3.79	4.00	0.09
Total error	5.69		
Total (corr.)	255.727		

Where F-ratio is the quotient of the middle squares of the data and *p*-value is the minimum statistical significance level.

**Table 3:** Different residual biomasses used in the removal of Ni (II).

Biomass	Adsorption Capacity (mg/g)	Reference
Alginate of *Sargassum filipendula*	12.62	[35]
NaOH-modified rice husks	26.60	[3]
Rice husk biochar	7.6	[36]
*Sargassum* sp.	1.23	[37]
Pumpkin waste	18.7	[38]
Clay	0.80	[39]
Smectite	0.66	
Goethite	0.78	
Birnese	0.71	
*Yersinibactinia*	2.00	[40]
Coconut shells	5.45	[16]
*Elaeis guineensis*	13.48	Present study

**Table 4:** Adjustment parameters for Ni (II) adsorption by non-linear adjustment.

Model	Parameter	Value
Adams–Bohart	*K_AB_* (L/min × mg)	4.3499 × 10^−5^
	N_0_ (mg/L)	1.54 × 10^4^
	Error	2.086 × 10^−1^
	R^2^	0.0165
Thomas	*K_th_* (mL/min × mg)	0.0840
	*q*_0_ (mg/g)	21.8716
	Error^2^	1.687 × 10^−1^
	R^2^	0.4053
Yoon–Nelson	*K_YN_* (min^−1^)	0.0084
	*t* (min)	212.6942
	Error^2^	1.687 × 10
	R^2^	0.4053
Dose–response	a	0.9821
	*q*_0_ (mg/g)	17.9882
	Error^2^	6.064 × 10^−2^
	R^2^	0.8456

## Data Availability

The data that support the findings of this study are available from the corresponding author upon reasonable request.
